# Epilepsy Is Associated With Dysregulation of Long Non-coding RNAs in the Peripheral Blood

**DOI:** 10.3389/fmolb.2019.00113

**Published:** 2019-10-23

**Authors:** Fatemeh Hashemian, Soudeh Ghafouri-Fard, Shahram Arsang-Jang, Sara Mirzajani, Hamid Fallah, Jafar Mehvari Habibabadi, Arezou Sayad, Mohammad Taheri

**Affiliations:** ^1^Department of Medical Genetics, Shahid Beheshti University of Medical Sciences, Tehran, Iran; ^2^Clinical Research Development Center (CRDU), Qom University of Medical Sciences, Qom, Iran; ^3^Isfahan Neuroscience Research Center, Isfahan University of Medical Sciences, Isfahan, Iran; ^4^Urogenital Stem Cell Research Center, Shahid Beheshti University of Medical Sciences, Tehran, Iran

**Keywords:** epilepsy, lncRNA, *HOXA-AS2*, *SPRY4-IT1*, *MEG3*, *LINC-ROR*

## Abstract

**Background:** Long non-coding RNAs (lncRNAs) are a group of functional transcripts that are not translated to proteins. Recent investigations have underscored their role in the pathogenesis of neurodevelopmental disorders.

**Methods:** In the current investigation, we quantified expression levels of four lncRNAs (*HOXA-AS2, SPRY4-IT1, MEG3*, and *LINC-ROR*) in peripheral blood of epileptic patients and normal controls.

**Results:** Expression of *HOXA-AS2* was significantly higher in patients compared with controls (Posterior beta = 1.982, *P* = 0.001). We detected interaction effects of gender on expression of *HOXA-AS2* (*P* = 0.012). Further analyses showed over-expression of *HOXA-AS2* in male patients compared with male controls (*P* = 0.003), in spite of similar levels of expression between female cases and female controls (*P* = 0.77). Expression of *SPRY4-IT1* was higher in total patients compared with total controls (Posterior beta = 1.27, *P* = 0.02). Such difference was only observed between male patients and male controls when dividing study participants based on their gender (*P* = 0.012). There was no significant difference in expression of *MEG3* and *LINC-ROR* between patients and controls.

**Conclusion:** Expression levels of all lncRNAs were correlated with each other with r values ranging from 0.61 to 0.76 (*P* < 0.0001). However, expressions of none of lncRNAs were correlated with age of study participants. The current data implies a putative role for two lncRNAs in the pathogenesis of epilepsy and warrants future functional studies to verify the observed association.

## Introduction

The role of long non-coding RNAs (lncRNAs) has been recently assessed in human diseases such as cancer and neurodevelopmental disorders (Shao and Chen, 2017; Sanchez Calle et al., 2018; Mazdeh et al., 2019). Epilepsy is among neurodevelopmental disorders in which lncRNAs might be involved (Shao and Chen, 2017). This chronic disorder is characterized by the presence of periodic unprovoked seizures and affects over 50 million individuals globally (de Boer et al., 2008). Although a number of anti-epileptic drugs (AED) have been approved for clinical use, none of them have attempted to influence the pathologic process of epilepsy (Shao and Chen, 2017). Thus, new methods for treatment of this disorder are highly appreciated. A prerequisite for development of such modalities is clarification of the pathogenesis of epilepsy. A previous microarray study in animal models has identified hundreds of aberrantly expressed lncRNAs in epileptic animals (Lee et al., 2015). Neurogenesis, production of neurotransmitter, passage of ions through cellular membranes, and synaptic plasticity are among mechanisms which are influenced by lncRNAs (Ng et al., 2013). Moreover, lncRNAs regulate several steps of neurogenesis during embryonic development whose abnormalities might be involved in the epilepsy (Mercer et al., 2010). Based on the above mentioned studies, we focused on a number of lncRNAs with putative roles in the epileptogenic process. The lncRNA *HOXA Cluster Antisense RNA 2* (*HOXA-AS2*) modulates the expression of *SCN3A* (Wu et al., 2019), an acknowledged gene in infantile epileptic encephalopathy (Zaman et al., 2018). Moreover, this antisense RNA is transcribed from *HOXA* cluster which contains genes being expressed in several regions of the developing brain (Nolte and Krumlauf, 2007). A recent study has also highlighted the role of aberrant DNA methylation of the *HOXA* gene cluster in the pathogenesis of Alzheimer's disease (Smith et al., 2018). *SPRY4 intronic transcript 1* (*SPRY4-IT1*) has a role in regulation of expression of estrogen-related receptor α (ERRα) (Yu et al., 2017), an orphan receptor with extensive expression in brain (Saito and Cui, 2018). The *maternally expressed gene 3* (*MEG3*) in highly expressed in brain and contributes in neuronal cell damage caused by subarachnoid hemorrhage through suppression of the PI3K/Akt pathway (Liang et al., 2018). The PI3K/Akt pathway has a protective role against epileptic seizure through enhancement of astrocyte proliferation and survival (Cao et al., 2018). *Long Intergenic Non-Protein Coding RNA, Regulator Of Reprogramming* (*LINC-ROR*) contributes in stem cell pluripotency (Loewer et al., 2010). In the current study, we measured expression of *HOXA-AS2, SPRY4-IT1, MEG3*, and *LINC-ROR* in the peripheral blood of epileptic patients and normal individuals to appraise their contribution in epileptogenic processes.

## Materials and Methods

### Study Participants

Totally, 40 epileptic patients and 40 normal individual were recruited. All patients had juvenile myoclonic epilepsy. All of them were taking valproic acid (between 3 and 18 months). Patients had no seizure attack throughout 6 months beforehand. None of them experienced febrile seizures. Diagnosis was based on electroencephalogram (EEG) and brain magnetic resonance imaging (MRI) [diffusion weighted (DW), T1, T2, and gradient eco images]. The study was approved by the ethics committee of Shahid Beheshti University of Medical Sciences. Informed consent forms were signed by all participants and the parents/Legally Authorized Representative of participants who were included in the study. Control group was chosen from individual who had no neurological, psychiatric or systemic disorder.

### Expression Assays

Five milliliters of peripheral blood was gathered from patients and control individuals in tubes containing 5 mM EDTA. Total RNA was extracted from blood specimens using Hybrid-RTM blood RNA extraction Kit (GeneAll, Seoul, South Korea). Next, the appropriateness of extracted RNA for additional phases of expression assay was judged using NanoDrop equipment (Thermo Scientific, MA, USA). First strand cDNA was produced using the OneStep RT-PCR Series Kit (BioFact™, Seoul, South Korea) according to company guidelines. Transcript levels of lncRNAs were quantified by using RealQ Plus 2 × PCR Master Mix Green Without ROX™ PCR Master Mix (Ampliqon, Odense, Denmark) in StepOnePlus™ RealTime PCR System (Applied Biosystems, Foster city, CA, USA). *B2M* gene was used as normalizer. Detailed information of primers is demonstrated in Table 1.

**Table 1:** Detailed information of primers.

**Primer name**	**Sequence**	**Primer length**	**PCR product length**
MEG3-F	TGGCATAGAGGAGGTGAT	18	111
MEG3-R	GGAGTGCTGTTGGAGAATA	19	
SPRY4-IT1-F	AGCCACATAAATTCAGCAGA	20	115
SPRY4-IT1-R	GATGTAGGATTCCTTTCA	18	
HOXA-AS2-F	CCCGTAGGAAGAACCGATGA	20	70
HOXA-AS2-R	TTTAGGCCTTCGCAGACAGC	20	
Linc-ROR-F	TATAATGAGATACCACCTTA	20	170
Linc-ROR-R	AGGAACTGTCATACCGTTTC	20	
B2M-F	AGATGAGTATGCCTGCCGTG	20	105
B2M-R	GCGGCATCTTCAAACCTCCA	20	

### Statistical Analyses

Analyses were performed in R software version 3.3.2. The differences in expression of lncRNAs between epileptic patients and normal individuals were assessed using Bayesian estimation. Normal distribution was assumed for parameters with 200,000 iterations. Spearman correlation test was applied for assessment correlation between lncRNAs expression amount as well as lncRNA expression and age. *P* <0.05 were regarded as significant.

## Results

### Detailed Data of Study Participants

The available information on study participants is presented in Table 2.

**Table 2:** Data of study participants.

**Variables**	**Patients**	**Controls**
Female/male [no. (%)]	25 (62.5%) / 15(50%)	22 (55%) / 18 (45%)
Age (mean ± SD, Y)	36.66 ± 2.8	34.06 ± 1.9
Age range (Y)	21–58	23–62
Age at onset (mean ± SD, Y)	28 ± 8.6	–
Disease duration (mean ± SD, Y)	8.18 ± 4.1	–

### Expression Assays

**Figure 1:**
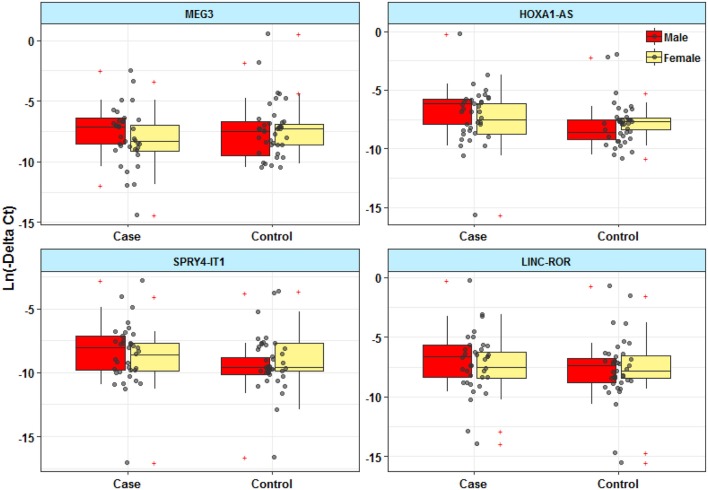
Expression levels of lncRNAs in peripheral blood of epileptic patients and healthy subjects.

Expressions of *HOXA-AS2* and *SPRY4-IT1* were significantly different between male patients and male controls (Figure 1). However, there was no significant difference in expression of *MEG3* and *LINC-ROR* between patients and controls.

Table 3 shows the detailed results of Bayesian Regression model for comparison of expression levels of lncRNAs between epileptic patients and healthy subjects. Expression of *HOXA-AS2* was significantly higher in patients compared with controls (Posterior beta = 1.982, *P* = 0.001). We detected interaction effects of gender on expression of *HOXA-AS2* (*P* = 0.012). Further analyses showed over-expression of *HOXA-AS2* in male patients compared with male controls (*P* = 0.003), in spite of similar levels of expression between female cases and female controls (*P* = 0.77).

**Table 3:** Results of Bayesian Regression model for comparison of expression levels of lncRNAs between epileptic patients and healthy subjects with adjusting the effects of age and gender.

		***MEG3***				***HOXA-AS2***				***SPRY4-IT1***				***LINC-ROR***			
**Groups**	**Variables**	**Posterior beta of RE**	**SE**	***P*-value**	**95% CrI for ER**	**Posterior beta of RE**	**SE**	***P*-value**	**95% CrI for ER**	**Posterior beta of RE**	**SE**	***P*-value**	**95% CrI for ER**	**Posterior beta of RE**	**SE**	***P*-value**	**95% CrI for ER**
Total	Group	0.333	0.7	0.447	[−1.01, 1.81]	1.928	0.6	0.001	[0.68, 3.01]	1.272	0.42	0.02	[0.52, 2.16]	1.032	0.72	0.439	[−0.39, 2.41]
	Age	0.464	0.62	0.434	[−0.73, 1.71]	0.64	0.49	0.13	[−0.29, 1.57]	0.231	0.53	0.788	[−0.75, 1.41]	−0.105	0.65	0.53	[−1.37, 1.19]
	Gender	0.045	0.03	0.207	[−0.01, 0.1]	0.015	0.03	0.583	[−0.04, 0.06]	−0.045	0.02	0.099	[−0.09, 0]	−0.006	0.03	0.66	[−0.07, 0.06]
	Group* Gender	−1.047	0.91	0.159	[−2.87, 0.7]	−1.684	0.81	0.012	[−3.23, −0.12]	−0.688	0.9	0.415	[−2.44, 1.1]	−0.538	0.95	0.697	[−2.43, 1.29]
Male	Group	0.338	0.74	0.55	[−1.13, 1.85]	1.888	0.67	0.003	[0.44, 3.07]	1.26	0.51	0.012	[0.27, 2.15]	0.964	0.71	0.113	[−0.43, 2.28]
	Age	0.028	0.04	0.423	[−0.05, 0.1]	−0.012	0.03	0.293	[−0.07, 0.05]	−0.043	0.03	0.364	[−0.1, 0.01]	−0.053	0.03	0.151	[−0.13, 0.01]
Female	Group	−0.774	0.58	0.689	[−1.97, 0.32]	0.31	0.54	0.775	[−0.72, 1.43]	0.466	0.65	0.21	[−0.83, 1.65]	0.322	0.66	0.584	[−1.01, 1.59]
	Age	0.062	0.05	0.22	[−0.04, 0.15]	0.056	0.04	0.262	[−0.03, 0.14]	−0.044	0.04	0.34	[−0.12, 0.04]	0.055	0.04	0.371	[−0.03, 0.14]

RE, relative expression; SE, standard errors; CrI, credible intervals; P-values are estimated from Frequentist method. *Interaction.

Expression of *SPRY4-IT1* was higher in total patients compared with total controls (Posterior beta = 1.27, *P* = 0.02). Such difference was only observed between male patients and male controls when dividing study participants based on their gender (*P* = 0.012).

### Correlations Between Expression Levels of lncRNAs

**Figure 2:**
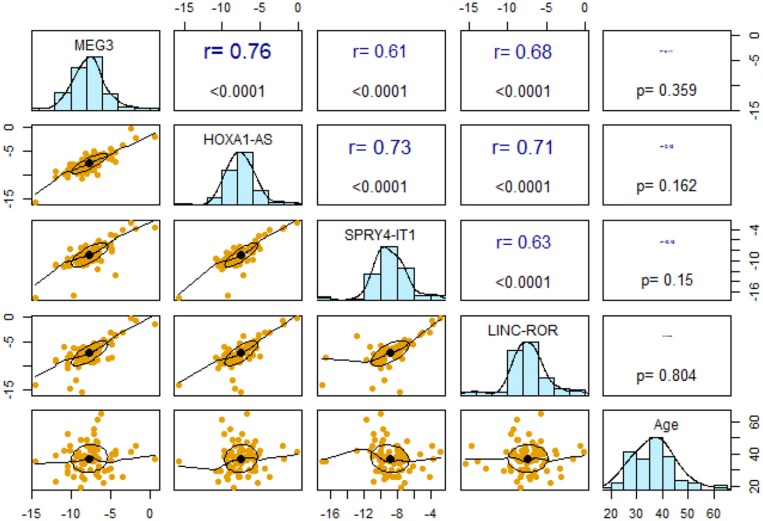
Pairwise correlations between expression levels of lncRNAs and their correlation with age.

Expression levels of all lncRNAs were correlated with each other with r values ranging from 0.61 to 0.76 (*P* <0.0001). However, expressions of none of lncRNAs were correlated with age of study participants (Figure 2).

Subsequently, we repeated correlation analyses in distinct gender-based groups of cases and controls (Table 4). The *SPRY4-IT1/LINC-ROR* and *SPRY4-IT1/MEG3* pairwise correlations were insignificant in female controls.

**Table 4:** Correlations between expression levels of lncRNAs in distinct groups [Spearman correlation coefficients (*P*-values) are presented].

**Group**		**Age**	***MEG3***	***HOXA-AS2***	***SPRY4-IT1***	***LINC-ROR***
Male patients	Age	1				
	*MEG3*	−0.38 (0.131)				
	*HOXA-AS2*	−0.34 (0.1825)	0.93 (<0.0001)			
	*SPRY4-IT1*	−0.25 (0.3264)	0.9 (<0.0001)	0.93 (<0.0001)		
	*LINC-ROR*	−0.42 (0.0927)	0.93 (<0.0001)	0.89 (<0.0001)	0.93 (<0.0001)	1
Male controls	Age	1				
	*MEG3*	0.46 (0.0492)				
	*HOXA-AS2*	0.3 (0.2183)	0.66 (0.002)			
	*SPRY4-IT1*	−0.26 (0.2839)	0.47 (0.044)	0.63 (0.004)		
	*LINC-ROR*	−0.03 (0.8892)	0.55 (0.0157)	0.75 (0.0002)	0.69 (0.001)	1
Female patients	Age	1				
	*MEG3*	0.23 (0.3282)				
	*HOXA-AS2*	0.25 (0.2876)	0.9 (<0.0001)			
	*SPRY4-IT1*	−0.04 (0.8673)	0.78 (0.0001)	0.82 (<0.0001)		
	*LINC-ROR*	0.26 (0.2714)	0.7 (0.0006)	0.69 (0.0008)	0.57 (0.0081)	1
Female controls	Age	1				
	*MEG3*	−0.09 (0.7019)				
	*HOXA-AS2*	0.1 (0.6429)	0.73 (0.0001)			
	*SPRY4-IT1*	−0.36 (0.0968)	0.36 (0.1048)	0.48 (0.0251)		
	*LINC-ROR*	0.16 (0.4772)	0.59 (0.0042)	0.45 (0.0341)	0.4 (0.064)	1

**Figure 3:**
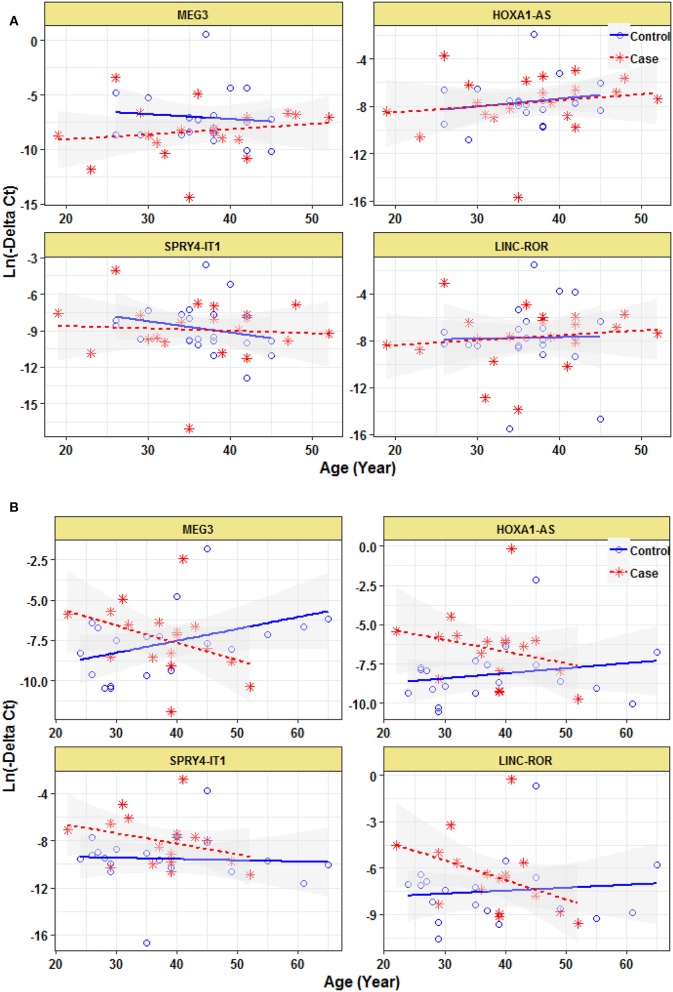
Correlation between expression levels of lncRNAs and age in female epileptic patients and healthy subjects **(A)** and male epileptic patients and healthy subjects **(B)**.

*Expression of none of lncRNAs were correlated with age even when study participants were divided based on their gender (Figure 3)*.

Expression of none of lncRNAs were correlated with age even when study participants were divided based on their gender (Figure 3).

## Discussion

Several lncRNAs have been formerly introduced as regulators of epilepsy-related processes such as neurogenesis, production of neurotransmitter, passage of ions through cellular membranes, and synaptic plasticity (Ng et al., 2013). In the current study, we quantified expression of four lncRNAs in epileptic patients. The functions of these selected lncRNAs were possibly related with epileptogenesis but their expressions have not been evaluated in epileptic patients before. We reported higher expressions of *HOXA-AS2* and *SPRY4-IT1* in male patients compared with male controls. However, expressions of these two lncRNAs were not different between female cases and female controls. A recent study has reported higher expression of both *HOXA-AS2* and *SPRY4-IT1* in patients with schizophrenia compared with controls. Contradictory to the present study, when authors assessed expression of these genes in gender-based manner, the differences in the expression of these lncRNAs were remarkable only among females (Fallah et al., 2019). These findings suggest different roles of these lncRNAs based on the gender of patients.

*HOXA-AS2* modulates the expression of *SCN3A* via interaction with hsa-miR-106a-5p (Wu et al., 2019). *SCN3A* gene has an acknowledged role in the epilepsy (Zaman et al., 2018) and hsa-miR-106a-5p participates in the pathogenesis of Alzheimer's disease (Patel et al., 2008). Notably, both gain- and loss-of-function mutations in *SCN3A* gene have been associated with seizure vulnerability (Chen et al., 2015; Lamar et al., 2017). So, the *HOXA-AS2*/ hsa-miR-106a-5p/ *SCN3A* axis is a putative target for future functional studies in epilepsy and a possible therapeutic target in this regard. The putative role of *SPRY4-IT1* in epileptogenesis might be exerted through its role in regulation of expression of ERRα (Yu et al., 2017). ERRα is an orphan nuclear receptor which has similar sequences with ERα. These two kinds of nuclear receptors have common transcriptional networks. Most notably, brain is a tissue with high expression of both ERα and ERRs (Saito and Cui, 2018). Although estrogen is not the intrinsic ligand for ERRα, the interaction between estrogen-signaling and ERRα might be facilitated through transcriptional regulation, or mutual binding on responsive elements, or via the regulation of estrogen production by aromatase (Saito and Cui, 2018). The observed gender-based differences in expressions of *HOXA-AS2* and *SPRY4-IT1* are in accordance with the results of former studies which highlighted the role of gender in modulation of the evolution of epilepsy. Some underlying mechanisms have also been clarified in the developing brain. Notably, gender-based dissimilarities in neuronal excitability, reaction to environmental stimulants, and epigenetic regulation of gene expression are among these mechanisms (Kight and McCarthy, 2014; Surguchov et al., 2017). In the case of *SPRY4-IT1*, the differences in relative abundance of ERRs in normal male and female brain might explain the observed gender-based differences as well. Alternatively, the presence of gender-specific transcription factors and epigenetic regulators might explain such differences between males and females.

Based on the high expression of *MEG3* in brain and its contribution in neuronal cell damage suppression of a brain-protective signaling pathway (Liang et al., 2018), we expected dysregulation of this lncRNA in epileptic patients. However, we did not detect any significant differences between study groups. So, we suggest that this lncRNA is not a probable contributor in epileptogenesis. Assessment of expression of this lncRNA in postmortem brain tissues might be needed for validation of this hypothesis.

In the current study, we did not have drug-naïve patients to assess the effects of valproic acid on expression of lncRNAs. A recent study has shown the effects of this AED on expression of certain genes associated with neuroprotection and neurotoxicity pathways in epileptic patients (Floriano-Sánchez et al., 2018). Both short and long-term treatment with this drug modulated mRNA levels of a several genes, and notably, returning most of the genes changed by the epileptic condition to a normal state (Floriano-Sánchez et al., 2018). Consequently, lack of difference in expression of *MEG3* and *LINC-ROR* between patients and controls in the current study might be explained by the effects of valproic acid on reverting their levels to normal state.

Notably, expression levels of all lncRNAs were correlated with each other in all subgroups except for lack of correlations between *SPRY4-IT1/LINC-ROR* and *SPRY4-IT1/MEG3* pairs in female controls. Such observation implies the presence of putative interaction network between them which might be affected by gender and disease status in some cases. However, expressions of none of lncRNAs were correlated with age of study participants which implies their independency from age or disease duration in patients. Taken together, the current data implies a putative role for two lncRNAs in the pathogenesis of epilepsy and warrants future functional studies to verify the observed association.

Our study has some limitations. The main weakness is that we assessed expression of a limited number of lncRNAs and limited amount of patients and, therefore, the conclusion about the significance of detected correlations is putative. Another limitation of our study is unavailability of drug-naïve patients.

## Data Availability Statement

This manuscript contains previously unpublished data. The name of the repository and accession number(s) are not available.

## Ethics Statement

The studies involving human participants were reviewed and approved by the ethics committee of Shahid Beheshti University of Medical Sciences. Informed consent forms were signed by all participants and the parents/Legally Authorized Representative of participants who were included in the study. The patients/participants provided their written informed consent to participate in this study.

## Author Contributions

FH and HF performed the experiment. AS and MT designed and supervised the study. SG-F wrote the manuscript and revised the draft. JM and SA-J analyzed the data. SM contributed in experiments setup. All the authors contributed equally and fully aware of the submission.

### Conflict of Interest

The authors declare that the research was conducted in the absence of any commercial or financial relationships that could be construed as a potential conflict of interest.
